# An overview of current phase 3 radiopharmaceutical therapy clinical trials

**DOI:** 10.3389/fmed.2025.1549676

**Published:** 2025-02-18

**Authors:** Nicolas Lepareur

**Affiliations:** ^1^Comprehensive Cancer Center Eugène Marquis, Rennes, France; ^2^Univ Rennes, Inrae, Inserm, Institut NUMECAN (Nutrition, Métabolismes et Cancer) – UMR_A 1341, UMR_S 1241, Rennes, France

**Keywords:** clinical trials, nuclear medicine, oncology, phase 3, radiopharmaceutical therapy

## Abstract

Over the past few years, radiopharmaceutical therapy has emerged as a groundbreaking therapeutic modality, taking advantage of the unique properties of radionuclides to deliver molecularly targeted therapy with high precision and transforming the landscape of precision oncology and personalized medicine. Its development reflects decades of advances in nuclear medicine, chemistry, and cancer biology. However, until recently, definitive clinical evidence was lacking to establish it into treatment plans, with few large randomized controlled clinical studies. The last two decades witnessed a paradigm shift, with three successful phase 3 studies which shed light on radiopharmaceutical therapy. This paper offers a brief overview of currently active phase 3 studies to highlight the dynamism and promise of this clinical domain, as well as the large variety of cancers being treated.

## 1 Introduction

The work of Maria Sklodowska Curie on radium and its applications in medicine led to the development, among other things, of radiopharmaceutical therapy (RPT). The field is expanding rapidly, thanks to advances in molecular biology, radiochemistry and computer science, enabling RPT to be used more and more routinely. RPT with iodine-131 has been used for over 80 years in thyroid pathologies. It simply uses the metabolic pathway of iodine in the thyroid cell, in which iodine-131 will remain and deliver its electrons, destroying the thyroid cells. Radioactivity is now increasingly delivered to a cellular target using radiopharmaceuticals that are as specific as possible, such as radiolabeled antibodies and peptides (1). For a long time, clinical studies evaluating the potential of therapeutic radiopharmaceuticals for cancer patients’ management were limited to small proof of concept studies, seldom reaching large, randomized phase 3 trials. Therefore, clinical evidence was lacking for their successful implementation, and radiopharmaceutical continued to be seen as a last resort treatment, available only in a small number of institutes. The last decade however saw three consecutive phase 3 trials that have represented a leap forward in the recognition of this therapeutic modality, the ALSYMPCA, NETTER-1, and VISION studies, eventually leading to the approval of, respectively, [^223^Ra]RaCl_2_ (Xofigo®), [^177^Lu]Lu-DOTATATE (Lutathera®) and [^177^Lu]Lu-PSMA-617 (Pluvicto®) (2–4). Now, with an increasing body of evidence and a growing implication of the pharmaceutical industry, phase 3 studies with therapeutic radiopharmaceuticals are becoming more common and several of them are currently underway. There are, at the present day, 34 active phase 3 trials, either expanding indications for already approved radiopharmaceuticals or evaluating new ones, in a variety of cancer types, as can be seen on Supplementary Table 1. In this paper, we aimed to provide an insight into these studies using RPT, based on an analysis of those phase 3 trials listed on clinicaltrials.gov.

**Figure 1:**
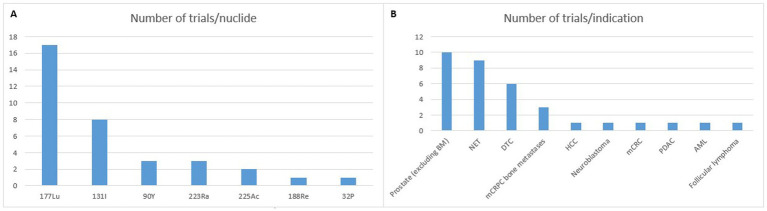
Phase 3 trials registered on clinicaltrials.gov (accessed 09/30/2024). BM: bone metastases; NET: neuroendocrine tumor; DTC: differentiated thyroid cancer; mCRPC: metastatic castration resistant prostate cancer; HCC: hepatocellular carcinoma; mCRC: metastatic colorectal cancer; PDAC: pancreatic ductal adenocarcinoma; AML: acute myeloid leukemia.

The most common targeted cancer types are neuroendocrine tumors, as well as prostate and thyroid carcinomas, representing nearly three-quarters of current studies (see Figure 1). Other indications include solid tumors, for instance hepatocellular carcinoma or liver metastases of colorectal cancer, as well as hematological malignancies, such as lymphomas and leukemia. Since the latter involve cells circulating throughout the bloodstream or within bone marrow, they are more challenging for safe and effective targeting than locally defined – and sometimes confined – solid tumors. Trial design might also be easier with solid tumors, thanks to the possibility to monitor tumor size via imaging techniques, while hematological ones require more complex biomarkers. Industrial considerations should not be overlooked, with a much higher incidence (and thus potential market) for solid tumors and a stronger competition with other treatment modalities in hematology. Therefore, current focus has been primarily in solid tumors.

Thanks to its interesting chemical and nuclear properties, lutetium-177 (t_1/2_ = 6.7 days; E_βmax_ = 0.498 MeV, *γ* emissions of 0.208 and 0.113 MeV) has become increasingly popular over the last couple of decades. It now appears as the current “gold standard” for RPT (5). In addition to the fact that the last two therapeutic radiopharmaceuticals being granted FDA and EMA approval were labeled with this nuclide, the huge majority of RPT clinical trials make use of this nuclide, with more than 200 studies currently underway. With regards to present-day phase 3 studies, 17 are using ^177^Lu (see Figure 1). Other radionuclides currently in use are essentially also *β*^−^-emitters (^131^I, ^90^Y, ^188^Re, and ^32^P). But *α*-particle emitters, such as ^223^Ra and ^225^Ac are gaining widespread interest. The use of the former remains limited to its chloride form for bone-pain palliation while the latter is increasingly being investigated as a replacement for ^177^Lu (6).

## 2 Clinical trials in neuroendocrine tumors

Neuroendocrine tumors overexpress somatostatin receptors, making them attractive targets. Numerous radiolabeled somatostatin analogs have been developed both for imaging and therapy (7). Among them, [^177^Lu]Lu-DOTATOC (with an EMA orphan drug designation) and [^177^Lu]Lu-DOTATATE have demonstrated their clinical usefulness for the management of patients suffering from gastroenteropancreatic neuroendocrine tumors (GEP-NETs). Following the NETTER-1 phase 3 trial, the latter was granted approval in inoperable or metastatic, progressive, well-differentiated (G1 and G2) GEP-NETs with positive sstr2 expression. Despite impressive results, there is nonetheless room for optimization, since all patients do not respond and, at longer follow-up, overall survival (OS) did not meet statistical significance versus high dose octreotide LAR (8). It is thus necessary to increase potency and efficacy, while individualizing the selection of patients to those most likely to respond to treatment. Similar phase 3 studies, comparing [^177^Lu]Lu-DOTATATE (with or without combination with standard dose octreotide LAR) and high dose (60 mg) octreotide LAR in G1/G2 GEP-NETs are currently running in China (**NCT05459844**, **NCT05884255**). Another study, COMPETE (**NCT03049189**), compared [^177^Lu]Lu-DOTATOC with everolimus, an mTOR inhibitor approved for the treatment of GEP-NETs. 309 patients have been enrolled and recruitment is now over. Differences in OS and progression-free survival (PFS) will be compared for a 30-month duration.

As opposed to external beam radiotherapy, dose distribution in RPT is highly heterogeneous at the microscopic tissue levels, with a dose distribution that can vary considerably depending on the patient, the regions of accumulation and retention of the radiotracer. Therefore, administering the same amount of radioactivity might not lead to the same absorbed dose to different patients. Initial studies have demonstrated personalization was feasible, allowing increased response rates without impairing tolerance (9). A Swedish multicenter randomized study (START-NET, **NCT05387603**) is now comparing standard 4 cycles of 7.5 GBq [^177^Lu]Lu-DOTATOC versus personalized treatment based on dual imaging ([^68^Ga]Ga-DOTATOC and [^18^F]-FDG) in terms of safety, efficacy and treatment cost. Replacement of the *β*^−^-emitting ^177^Lu with an *α*-particle emitter has been proposed as a mean to improve therapeutic efficacy, with preclinical and clinical data supporting this hypothesis (10). In particular, [^225^Ac]Ac-DOTATATE (RYZ101®) is currently evaluated in a phase 1b/3 study (ACTION-I, **NCT05477576**) in patients that have progressed following a previous treatment with ^177^Lu-labeled somatostatin analogs. The first part of this study will determine the safety, pharmacokinetics and recommended phase 3 dose (RP3D), while the second part will compare the radiopharmaceutical with the standard of care (everolimus, sunitinib, octreotide, or lanreotide) based on the RP3D determined beforehand.

Several studies are also aimed at expanding the clinical indications of ^177^Lu-labeled somatostatin analogs, supported by the results of preliminary studies (11). NETTER-2 (**NCT03972488**) and COMPOSE (**NCT04919226**) trials, respectively with [^177^Lu]Lu-DOTATATE and [^177^Lu]Lu-DOTATOC, ambition to evaluate the efficacy and safety of this treatment modality in patients with grade 2 and 3 GEP-NETs. Primary analysis of NETTER-2 seems to indicate it could be considered a new standard of care in first-line therapy in this population (12). Since sstr2 expression is not limited to GEP-NETs, investigation of the efficacy and safety of ^177^Lu-labeled somatostatin analogs is also performed in neuroendocrine neoplasms other than grade G1/G2 GEP-NETs (**NCT06398444**) and in lung and thymic carcinoids (**NCT05918302**).

## 3 Clinical trials in prostate cancer

Until recently, prostate cancer management with RPT was essentially limited to bone metastases pain palliation. The *α*-emitting [^223^Ra]RaCl_2_ was the first radiopharmaceutical to provide survival benefit for patients with metastatic castration-resistant prostate cancer (mCRPC), beyond sole pain relief (2). A retrospective phase 2 study using [^188^Re]Re-HEDP also showed an increased life expectancy (13). The randomized RaRe study (**NCT03458559**) in Netherlands aims to compare those two radiopharmaceuticals, though current status of this trial is unknown. The integration of RPT with other treatments, such as hormonotherapy or chemotherapy, shows promise for managing mCRPC. But determining optimal combination and sequencing requires further investigation, with various clinical trials investigating new therapeutic approaches (14). There are in particular two international multicenter randomized studies investigating such combinations. PEACE III trial (**NCT02194842**) is investigating the combination of [^223^Ra]RaCl_2_ with enzalutamide, a non-steroidal antiandrogen, while DORA trial (**NCT03574571**) is investigating its combination with docetaxel, a microtubule disruptor. However, with the emergence of radioligand therapy with PSMA-targeting radiopharmaceuticals, the question of the future usefulness of these “bone-seeking only” radiopharmaceuticals is worth asking.

The introduction of ^177^Lu-labeled PSMA-inhibitors has revolutionized the management of prostate cancer in recent years (15). Two prominent compounds, PSMA-617 and PSMA-I&T have demonstrated similar behavior (16). While the first has been EMA-and FDA-approved, the second is presently used in compassionate setting and the subject of two active phase 3 studies in mCRPC patients (**NCT04647526**, **NCT05204927**). Recruitment for these studies is now complete and results are awaited for potential approval. [^177^Lu]Lu-PSMA-617 is also the subject of numerous clinical trials, among which 5 are phase 3 studies (17). These aim to establish the feasibility to treat patients in earlier stages of the disease, in metastatic hormone-sensitive (**NCT04720157**, **NCT06496581**, **NCT06320067**) or oligometastatic prostate cancers (**NCT05939414**). Given the epidemiology of this pathology, large cohorts are expected. For instance, within PSMAddition study (**NCT04720157**), 1,148 patients have already been enrolled, while 8,000 are expected for STAMPEDE2 study (**NCT06320067**). Another study, PSMAfore (**NCT04689828**), aims to assess the superiority of [^177^Lu]Lu-PSMA-617 over a change of androgen receptor-directed therapy (ARDT) treatment in prolonging PFS, as measured radiographically (rPFS), and improving OS for mCRPC participants.

Since the seminal paper by Kratochwil *et al.* establishing its feasibility, ^225^Ac-labeled PSMA ligands have gained momentum (18). Several ongoing trials are currently investigating the potential of ^225^Ac to replace ^177^Lu, including a large retrospective study (WARMTH Act) providing evidence to support its use (19). AlphaBreak trial (**NCT06402331**) is a randomized multicenter phase 2/3 in which one of the objectives is to find the optimal dose regimen with [^225^Ac]Ac-PSMA-I&T in patients previously treated with ^177^Lu-PSMA radioligand therapy. In addition to small molecule PSMA-inhibitors, monoclonal antibodies targeting PSMA also look promising. Several studies using [^177^Lu]Lu-rosopatamab (^177^Lu-J591) evidenced some therapeutic efficacy, justifying the set-up of two multinational randomized trials (**NCT04876651**, **NCT06520345**) both comparing the combination of this radiolabeled mAb with standard of care versus standard of care alone (20). Of note, a phase 2 study with [^225^Ac]Ac-rosopatamab has recently started recruitment (CONVERGE-01 trial).

## 4 Clinical trials in thyroid cancer

Differentiated thyroid cancer (DTC), comprising follicular and papillary variants, represents 90% of thyroid cancers. Standard procedure for the management of DTC is surgery (either total thyroidectomy or unilobar lobectomy), often followed by radioiodine administration to ablate residual normal thyroid tissue and treat persistent or recurrent disease (21). Though radioactive iodine ablation (RAI) with Na[^131^I]I is a well-established treatment procedure, with millions of patients treated, there are still investigations to assess its efficacy and safety. For instance, it has been reported that RAI might impact fertility because of deleterious effects on ovarian reserve (22). Use of metformin, an oral antihyperglycemic drug, might help to maintain an acceptable level of ovary follicle number, as demonstrated in women with polycystic ovary syndrome. The investigation of the effect of this drug in combination with RAI is the subject of the METHYR trial (**NCT05468554**). A legitimate question might thus be whether or not RAI is necessary for patients with low-or intermediate-risk DTC. Several large randomized trials have been searching to determine in which cases it might be deemed useful (**NCT01398085**, **NCT01837745**, **NCT04290663**). 5-years follow-up results from the ESTIMABL2 trial (**NCT01837745**), conducted across 35 centers of the French Endocan-TuThyRef network, have just been published, concluding simple surveillance was non-inferior to RAI in patients with low-risk well differentiated thyroid carcinoma after total thyroidectomy (23). 94.8% of patients had no events (either functional, structural or biological) in the radioiodine group vs. 93.2% in the no-radioiodine group. These results are consistent with intermediate ones from IoN study (**NCT01398085**), considering only structural events.

## 5 Clinical trials in other solid tumors

RPT is increasingly being used in the treatment of solid tumors, with numerous ongoing clinical trials in multiple tumor types beside NETs and mCRPCs (24). Neuroblastoma is a common pediatric cancer that develops from immature nerve cells. Since 90% of neuroblastomas take up meta-iodobenzylguanidine (mIBG), a norepinephrine analog, [^131^I]mIBG has been used for years for neuroblastoma treatment and has been granted orphan designation in this indication by EMA, but advances are still needed. **NCT03126916** trial, sponsored by the Children’s Oncology Group in the USA, aims to evaluate whether event-free survival (EFS) of patients with newly diagnosed high-risk neuroblastoma is improved with the addition of [^131^I]mIBG or lorlatinib, an ALK inhibitor, to intensive therapy (chemotherapy + external beam radiotherapy). Previous studies demonstrated [^131^I]mIBG administration was feasible in this indication, laying the groundwork for this randomized trial (25).

Radiolabeled microspheres are a particular type of radiopharmaceuticals that have found wide use in digestive cancer treatment, in particular primary and secondary liver cancers, so-called radioembolization (26). Because microspheres are usually considered an active implantable medical device (they are marketed as such), clinical trials have been a long time coming, with the first phase 3 studies to be reported only in the 2010s, in hepatocellular carcinoma (HCC) and liver metastases of colorectal carcinoma (mCRC), with mixed results despite many encouraging results based on cohort and phase 2 studies. These particles are injected directly into the tissue to be treated or through a tumor-feeding artery, as a locoregional treatment or a form of brachytherapy, and their colloidal state prevents them from spreading beyond the injection site, since the particles are phagocytosed by the cells. When dealing with radioembolization, the term is essentially applied for yttrium-90 loaded microspheres, with two commercially available microspheres, either glass based (TheraSphere™) or resin based (SIR-Spheres®), differing in size, number of injected microspheres and activity per microsphere. Though they start to enter clinical guidelines, both are still the subject of phase 3 trials to definitely establish them as clinically useful treatment options. MANDARIN trial (**NCT05016245**), currently underway in China, aims to compare the efficacy and safety of ^90^Y-glass microspheres with conventional transarterial chemoembolization (cTACE), which remains the gold standard for intermediate HCC, in patients with inoperable HCC. This will represent a small study, since, with 92 patients already enrolled, recruitment is not active. A few years ago, an American randomized phase 2 study in 45 BCLC A or B patients concluded on the superiority of radioembolization over cTACE (27). A second phase 3 study, SIR-step trial (**NCT01895257**), of which status is currently unknown, employs resin-based ^90^Y-loaded microspheres as an adjuvant to maintenance chemotherapy with levoleucovorin and 5-FU (LV5FU2), to investigate if it has a benefit in terms of time to progression and survival in patients with dominant or exclusive and unresectable liver metastases from colorectal cancer (mCRC) controlled after 3–6 months of chemotherapy induction. This study should have reached completion by now, but no results have been published so far.

Additional radiolabeled microspheres are being explored, including holmium-166 microspheres and phosphorus-32 microspheres (28). A new device using ^32^P-microparticles (OncoSil™) implanted directly into the tumor has been recently developed, and used for the treatment of unresectable locally advanced pancreatic ductal adenocarcinoma (PDAC), which remains a cancer with a very grim prognosis. Following successful pilot studies, such as the PanCO trial (NCT03003078) (29), a small phase 2/3 cohort study, with all patients receiving the treatment (OncoSil™ + gemcitabine) is currently recruiting in one center, in Hong-Kong, with an estimated enrollment of 20 patients. Measured outcomes will be adverse events, disease control rates and survival (progression-free and overall).

## 6 Clinical trials in hematological malignancies

Radioimmunotherapy (RIT), the use of radiolabeled antibodies is a particularly attractive approach for hematologic malignancies thanks to the existence of many easily accessible and highly specific cell surface antigens that are not expressed on other tissues and the availability of a multitude of monoclonal antibodies (mAbs) specific to these antigens. The early 2000s saw the emergence and approval of two radiolabeled anti-CD20 mAbs, respectively labeled with iodine-131 (Bexxar®) and yttrium-90 (Zevalin®) (30). Despite commercial failure for these two radiopharmaceuticals, an Italian multicenter, open-label, randomized and controlled study comparing the efficacy of Zevalin® vs. autologous stem cell transplantation (ASCT) in patients with relapsed/refractory follicular lymphoma after second or third line chemotherapy supplemented with rituximab is still active (**NCT01827605**). Completion of the study was planned in 2024, with 159 patients recruited for an estimated original enrollment of 265. Previous studies have demonstrated that Zevalin® therapy is safe and effective in this indication, irrespective of prior treatment with rituximab (31).

Iomab-B is another ^131^I-radiolabeled antibody (apamistamab, an anti-CD45 mAb) used in multiple blood cancer indications (32), which is currently investigated in the SIERRA phase 3 study (**NCT02665065**), in patients with relapsed or refractory acute myeloid leukemia (AML). Interim results reported so far demonstrate a beneficial effect, with 92% 1-year survival and 69% 2-year survival, and a complete response achieved in 74.6% of evaluable patients. Based on those results, an FDA biologics license application was recently requested. FDA nonetheless requested an additional head-to-head randomized trial demonstrating an improvement in overall survival. Indeed, in SIERRA trial, circa 60% of patients crossed over from the control arm to the investigational arm, thus confounding OS results in the intention-to-treat population.

## 7 Conclusion

Radiopharmaceutical therapy has evolved significantly over the past decades. Increasingly, large, prospective, randomized and carefully designed trials are being conducted to establish its clinical utility and integrate it into treatment plans. Those trials aim to improve therapy efficacy while limiting toxicity in association with other therapies as well as to expand clinical indications, particularly earlier in the patient’s treatment course. In this respect, RPT has emerged as a very promising therapeutic option for a wide spectrum of cancers.

## Supplementary material

SUPPLEMENTARY TABLE 1 List of current RPT phase 3 trials.
