# Supplementary material for: An overview of current phase 3 radiopharmaceutical therapy clinical trials
Source: Front Med (Lausanne). 2025 Feb 18;12:1549676. doi: 10.3389/fmed.2025.1549676 (PMC11876376; doi:10.3389/fmed.2025.1549676)
Supplement: SUPPLEMENTARY TABLE 1 — List of current RPT phase 3 trials. [file Table_1.DOCX]

|  | **Study/Name** | **Therapeutic agent/Treatment** | **Radionuclide** | **Disease*** | **Primary outcome** | **Study Start (Actual)** | **Status** | **Estimated enrollment** | **Sponsor** | **Location**  **(number of sites)** |
| --- | --- | --- | --- | --- | --- | --- | --- | --- | --- | --- |
| **Neuroendocrine tumors** | NCT06398444 | **[^177^Lu]Lu-DOTATATE** vs. octreotide LAR | ^177^Lu | Advanced NET other than G1/2 GEP-NET* | ORR | 2024-06-01 | Not yet recruiting | 74 | Sinotau Pharmaceutical Group | China (1) |
|  | NCT05884255 | **[^177^Lu]Lu-DOTATATE** + octreotide LAR vs. octreotide LAR | ^177^Lu | Advanced GEP-NET | PFS | 2023-07-06 | Recruiting | 220 | Jiangsu HengRui Medicine Co., Ltd. | China (1) |
|  | NCT05459844 | **[^177^Lu]Lu-DOTATATE** vs. octreotide LAR | ^177^Lu | Grade 1/2 GEP-NET | PFS | 2022-08-31 | Active, not recruiting | 196 | Sinotau Pharmaceutical Group | China (26) |
|  | **NETTER-2**  NCT03972488 | **[^177^Lu]Lu-DOTATATE** vs. octreotide LAR | ^177^Lu | Grade 2/3 GEP-NET* | PFS | 2020-01-08 | Active, not recruiting | 226 | Advanced Accelerator Applications | Global (40) |
|  | **ACTION-1** NCT05477576 | **[^225^Ac]Ac- DOTATATE** vs. SoC | ^225^Ac | GEP-NET | RP3D; PFS | 2022-03-24 | Recruiting | 288 | RayzeBio, Inc. | Global (50) |
|  | **LEVEL**  NCT05918302 | **[^177^Lu]Lu-DOTATOC** vs. everolimus | ^177^Lu | Lung and thymus NET* | PFS | 2023-10-27 | Recruiting | 120 | Grupo Espanol de Tumores Neuroendocrinos | France, Italy, Spain (26) |
|  | **START-NET**  NCT05387603 | **[^177^Lu]Lu-DOTATOC** +/- capecitabin | ^177^Lu | NEN* | PFS | 2022-11-01 | Recruiting | 300 | Lund University Hospital | Sweden (4) |
|  | **COMPOSE**  NCT04919226 | **[^177^Lu]Lu-DOTATOC** vs. SoC | ^177^Lu | Grade 2/3 GEP-NET* | PFS | 2021-12-21 | Recruiting | 250 | ITM Solucin GmbH | Global (43) |
|  | **COMPETE**  NCT03049189 | **[^177^Lu]Lu-DOTATOC** vs. everolimus | ^177^Lu | GEP-NET | PFS | 2017-02-02 | Active, not recruiting | 309 | ITM Solucin GmbH | Global (52) |
| **Prostate cancer** | **PSMAfore**  NCT04689828 | **[^177^Lu]Lu-PSMA-617** vs. ARDT | ^177^Lu | mCRPC | rPFS | 2021-06-15 | Active, not recruiting | 469 | Novartis Pharmaceuticals | USA, Europe (72) |
|  | **PEACE6-Poor Responders**  NCT06496581 | **[^177^Lu]Lu-PSMA-617** + SoC vs. SoC | ^177^Lu | mHSPC* | OS and rPFS | 2024-08-01 | Not yet recruiting | 500 | UNICANCER | France (25) |
|  | **STAMPEDE2** NCT06320067 | **[^177^Lu]Lu-PSMA-617** vs. SABR vs. Niraparib vs. SoC | ^177^Lu | mHSPC* | OS and rPFS | 2024-06-11 | Recruiting | 8000 | University College, London | UK (4) |
|  | **PSMAddition**  NCT04720157 | **[^177^Lu]Lu-PSMA-617** + SoC vs. SoC | ^177^Lu | mHSPC* | rPFS | 2021-06-09 | Active, not recruiting | 1145 | Novartis Pharmaceuticals | Global (164) |
|  | **PSMA-DC**  NCT05939414 | **[^177^Lu]Lu-PSMA-617** + SBRT | ^177^Lu | Oligometastatic PC* | MFS | 2024-03-12 | Recruiting | 450 | Novartis Pharmaceuticals | Global (64) |
|  | **ECLIPSE**  NCT05204927 | **[^177^Lu]Lu-PSMA-I&T** vs. SoC | ^177^Lu | mCRPC | rPFS | 2022-02-14 | Active, not recruiting | 439 | Curium US LLC | USA, Europe (52) |
|  | **SPLASH**  NCT04647526 | **[^177^Lu]Lu-PSMA-I&T** vs. hormonotherapy | ^177^Lu | mCRPC | rPFS | 2021-02-25 | Active, not recruiting | 415 | POINT Biopharma | USA, Europe (54) |
|  | **AlphaBreak** NCT06402331 | **[^225^Ac]Ac-PSMA-I&T** | ^225^Ac | mCRPC | TEAEs and PSA50 response | 2024-03-05 | Recruiting | 60 | Fusion Pharmaceuticals Inc. | USA (6) |
|  | **ProstACT GLOBAL**  NCT06520345 | **[^177^Lu]Lu-DOTA-rosopatamab** + SoC vs. SoC | ^177^Lu | mCRPC | rPFS | 2024-07-26 | Recruiting | 430 | Telix Pharmaceuticals (Innovations) Pty Ltd | USA, Australia (7) |
|  | NCT04876651 | **[^177^Lu]Lu-DOTA-rosopatamab** + SoC vs. SoC | ^177^Lu | mCRPC | rPFS | 2023-08-29 | Recruiting | 392 | Telix Pharmaceuticals (Innovations) Pty Ltd | Australia, New Zealand (6) |
|  | NCT03574571 | Docetaxel +/- **[^223^Ra]RaCl_2_** | ^223^Ra | mCRPC | OS | 2018-06-19 | Recruiting | 738 | Memorial Sloan Kettering Cancer Center | Global (69) |
|  | **PEACE III**  NCT02194842 | Enzalutamide +/- **[^223^Ra]RaCl_2_** | ^223^Ra | mCRPC | rPFS | 2015-10 | Active, not recruiting | 446 | EORTC | Global (64) |
|  | **RaRe**  NCT03458559 | **[^223^Ra]RaCl_2_**/  **[^188^Re]Re-HEDP** | ^223^Ra/^188^Re | mCRPC | OS | 2018-05-16 | Unknown | 402 | Amsterdam UMC | Netherlands (1) |
| **Differentiated thyroid carcinoma** | **METHYR**  NCT05468554 | **[^131^I]INa** +/- metformin | ^131^I | DTC | Changes in AMH, Inhibin B and FSH levels due to the action of metformin | 2022-11-01 | Not yet recruiting | 160 | Medical University of Bialystok | Poland (1) |
|  | NCT04964284 | **[^131^I]INa** +/- rhTSH | ^131^I | DTC | Rate of successful postoperative thyroid ablation | 2021-10-06 | Recruiting | 328 | Suzhou Zelgen Biopharmaceuticals Co.,Ltd | China (1) |
|  | **RABITO**  NCT05142904 | **[^131^I]INa** vs. RFA | ^131^I | DTC | Hypothyroidism | 2021-11-17 | Recruiting | 232 | Rijnstate Hospital | Netherlands (14) |
|  | **INTERMEDIATE**  NCT04290663 | **[^131^I]INa** | ^131^I | DTC | Rate of patients with excellent tumoral response | 2020-03-02 | Recruiting | 476 | Centre Francois Baclesse | France (28) |
|  | **ESTIMABL2**  NCT01837745 | **[^131^I]INa** | ^131^I | DTC | Rate of patients without event at 3 years following randomization | 2013-05-13 | Active, not recruiting | 776 | Institut Gustave Roussy | France (35) |
|  | **IoN**  NCT01398085 | **[^131^I]INa** | ^131^I | DTC | Disease-free thyroid specific survival | 2012-05 | Active, not recruiting | 504 | University College, London | UK (32) |
| **Other solid tumors** | NCT03126916 | Intensive Therapy + **[^131^I]MIBG** or Lorlatinib | ^131^I | High-risk Neuroblastoma | EFS | 2018-05-14 | Active, not recruiting | 724 | Children's Oncology Group | USA, Canada (161) |
|  | **MANDARIN**  NCT05016245 | **^90^Y-glass microspheres** | ^90^Y | HCC | TTP and safety assessed within 60 days post-treatment | 2021-09-13 | Active, not recruiting | 92 | Boston Scientific Corporation | China (1) |
|  | **SIR-step**  NCT01895257 | **^90^Y-resin microspheres** + LV5FU2 | ^90^Y | CRC liver metastases | Time to progression | 2013-08 | Unknown | 162 | Universiteit Antwerpen | Belgium (10) |
|  | NCT05131776 | **OncoSil™** | ^32^P | Locally advanced PDAC | Adverse events | 2021-11-01 | Recruiting | 20 | Chinese University of Hong Kong | Hong Kong (1) |
| **Blood cancers** | NCT01827605 | **[^90^Y]Y-ibritumomab tiuxetan** | ^90^Y | Relapsed FL | PFS | 2012-01 | Active, not recruiting | 159 | Fondazione Italiana Linfomi - ETS | Italy (38) |
|  | **SIERRA**  NCT02665065 | **Iomab-B** vs. conventional care | ^131^I | AML | dCR | 2016-06 | Active, not recruiting | 153 | Actinium Pharmaceuticals | USA (24) |

***** Indication expansion; **AML**: acute myeloid leukemia; **ARDT**: androgen receptor-directed therapy; **CRC**: colorectal cancer; **dCR**: durable complete remission; **DTC**: differentiated thyroid cancer; **EFS**: event-free survival; **FL**: follicular lymphoma; **HCC**: hepatocellular carcinoma; **mCRPC**: metastatic castration resistant prostate cancer; **MFS**: metastasis free survival; **mHSPC**: Metastatic hormone-sensitive prostate cancer; **NEN**: neuroendocrine neoplasia; **(GEP-)NET**: (gastroenteropancreatic) neuroendocrine tumor; **ORR**: overall response rate; **OS**: overall survival; **PDAC**: pancreatic ductal adenocarcinoma; **PFS**: progression-free survival; **PSA50 response**: decline in PSA levels by at least 50%; **RFA**: radiofrequency ablation**; rhTSH**: recombinant human thyrotropin; **RP3D**: recommended phase 3 dose; **rPFS**: radiographic progression free survival; **SABR**: stereotactic ablative body radiotherapy; **SBRT**: stereotactic body radiation therapy; **SoC**: standard of care; **TEAE**: treatment-emergent adverse event; **TTP**: time to progressions
